# Supplementary material for: N-Glucosylation in Corynebacterium glutamicum with YdhE from Bacillus lichenformis
Source: Molecules. 2022 May 25;27(11):3405. doi: 10.3390/molecules27113405 (PMC9182490; doi:10.3390/molecules27113405)
Supplement: Supplementary file 1 [file molecules-27-03405-s001.zip › molecules-1733700-supplementary.pdf]

molecules

Supplementary Materials

N-Glucosylation in *Corynebacterium Glutamicum* with YdhE from *Bacillus lichenformis*

Obed Jackson Amoah [1,†], Hue Thi Nguyen [1,†] and Jae Kyung Sohng [1,2,*]

1 Department of Life Science and Biochemical Engineering, Sun Moon University, 70 Sunmoon-ro 221, Tangjeong-myeon, Asan-si, Chungnam 31460, Republic of Korea
2 Department of Pharmaceutical Engineering and Biotechnology, Sun Moon University, 70 Sunmoon-ro 221, Tangjeong-myeon, Asan-si, Chungnam 31460, Republic of Korea
† These authors are equally contributed.
* Correspondence: sohng@sunmoon.ac.kr, Tel: +82 (41) 530-2246, Fax: +82 (41) 530-8229

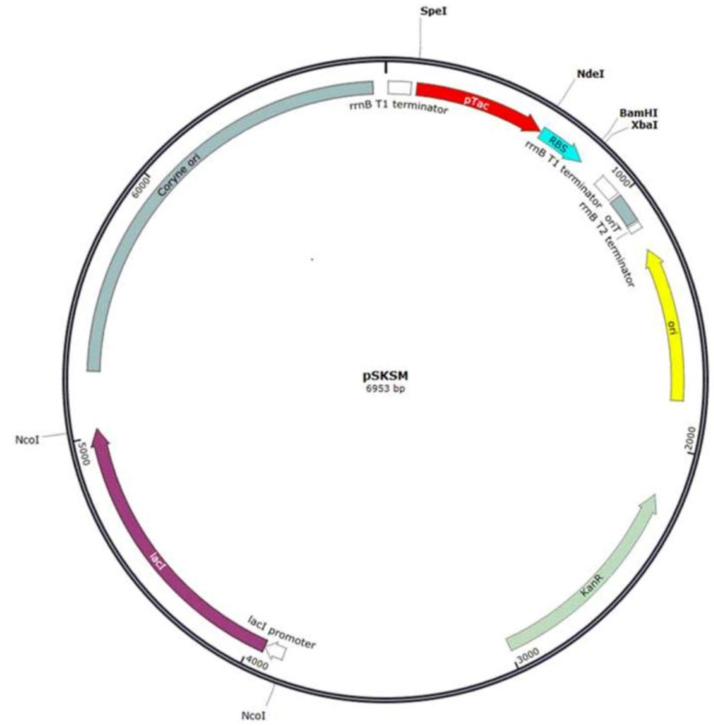

Figure S1. Map of pSKSM.

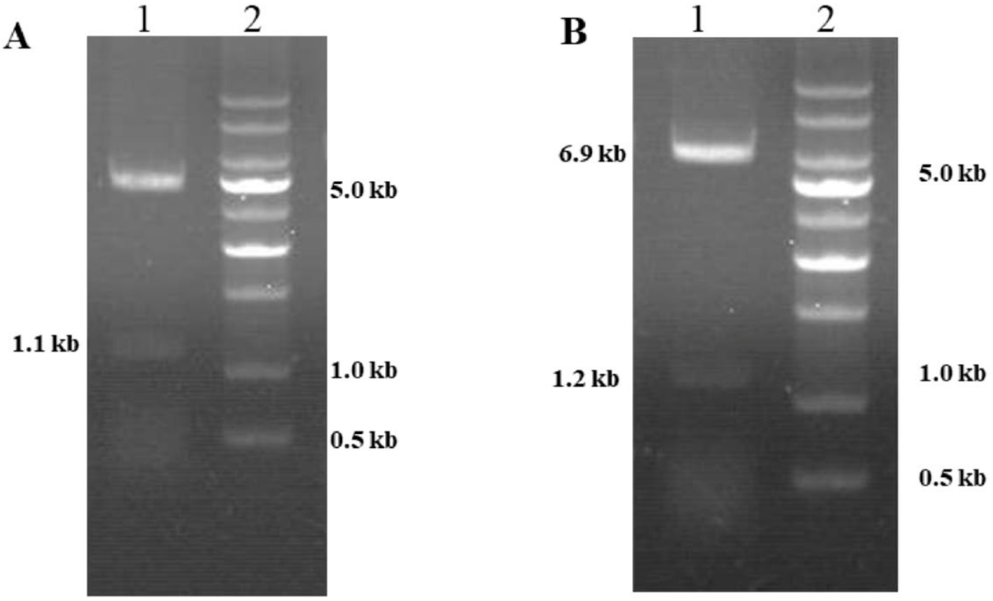

Figure S2. Cloning of CO−YdhE in pSKSM vector; (**A**) Lactose repressor (LacI) from pET32a(+) cloned into pSK003 vector harboring *tac* promoter (*pTac*) to form pSKSM vector; lane 1: LacI in pSK003; lane 2: DNA marker. (**B**) Glycosyltransferase *ydhE* (Codon-Optimized) cloned into pSKSM to form pSKSM−YdhE; lane 1: CO−YdhE in pSKSM; lane2: DNA marker.

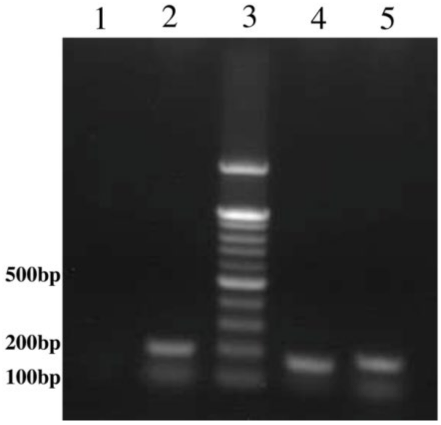

Figure S3. RT-PCR of CO-YdhE in *C. glutamicum* ; Lane 1; RT-PCR of CO-YdhE in *C. glutamicum* wild type ; Lane 2; RT-PCR of *Ncgl272* as housekeeping gene in *C. glutamicum* wild type (200 bp), Lane 3; DNA Marker, Lane 4; CO-YdhE (180 bp) in *C. glutamicum* harboring pSKSM–YdhE, Lane 5; *Ncgl272* (200 bp) in *C. glutamicum* harboring harboring pSKSM–YdhE.

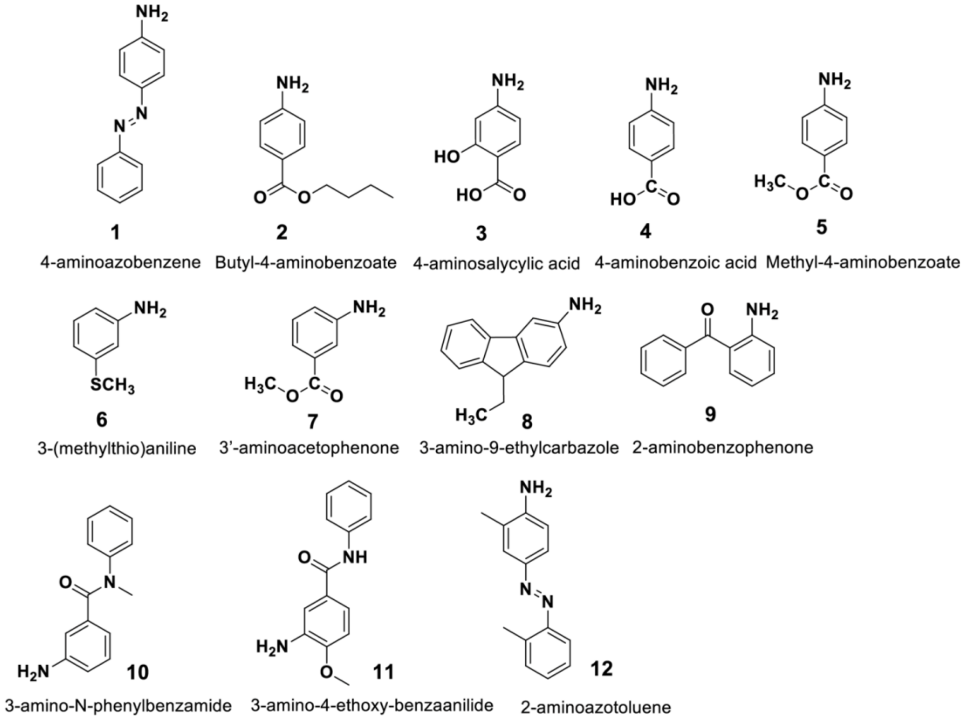

Figure S4. Structure of substrates used in this study.

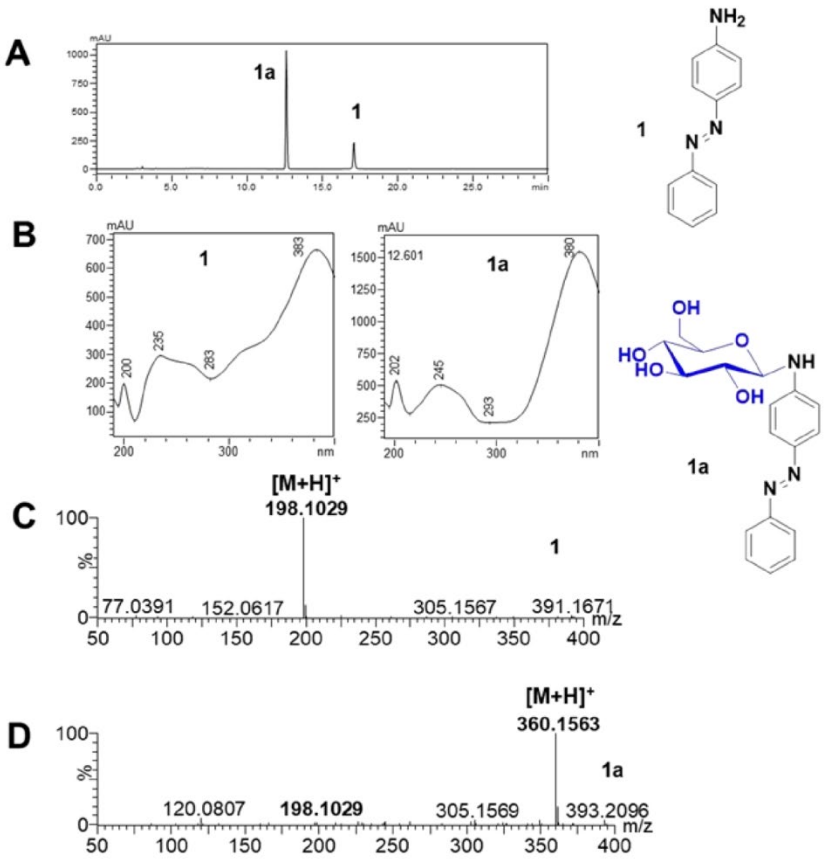

Figure S5. HPLC−PDA and HR−QTOF ESI/MS analysis conversion of 4-aminoazobenzene (**1**) as an aglycon acceptor of YdhE in *C. glutamicum*. (**A**) HPLC chromatogram; (**B**) UV spectra of **1** and glucosylated product **1a**; (**C**) Selected ion chromatogram at *m/z* 198.1029 for substrate **1** $[M+H]^+$ and (**D**) Selected ion chromatogram at *m/z* 360.1563 for glucosylated product of **1** $[M+H]^+$.

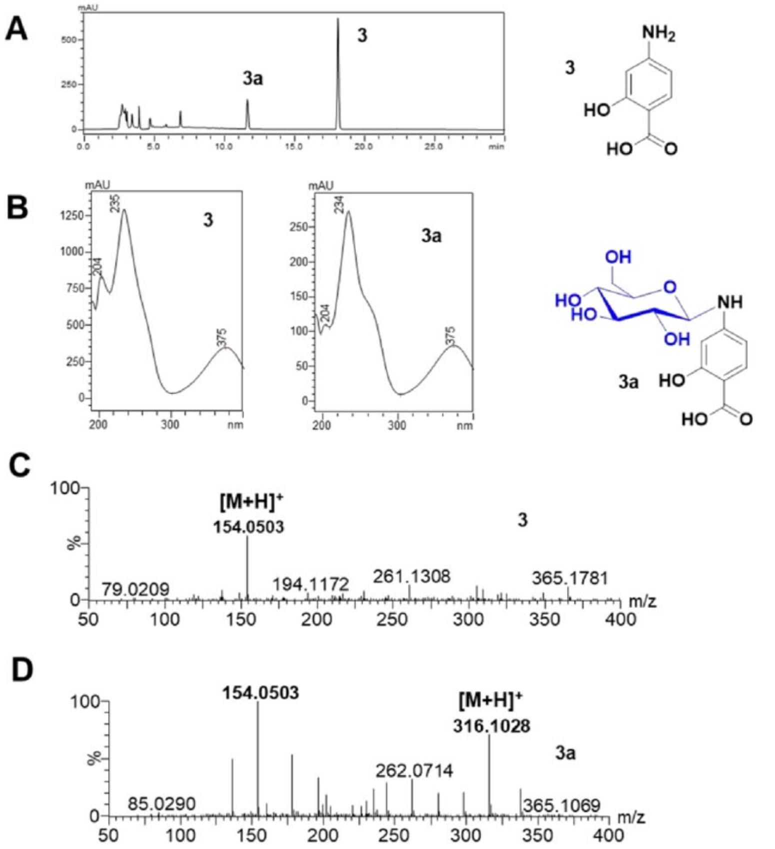

Figure S6. HPLC−PDA and HR−QTOF ESI/MS analysis conversion of 4-aminosalicylic acid (**3**) as an aglycon acceptor of YdhE in *C. glutamicum*. (**A**) HPLC chromatogram; (**B**) UV spectra of **3** and glucosylated product **3a**; (**C**) Selected ion chromatogram at *m/z* 154.0503 for substrate **3** $[M+H]^+$ and (**D**) Selected ion chromatogram at *m/z* 316.1028 for glucosylated product of **3** $[M+H]^+$.

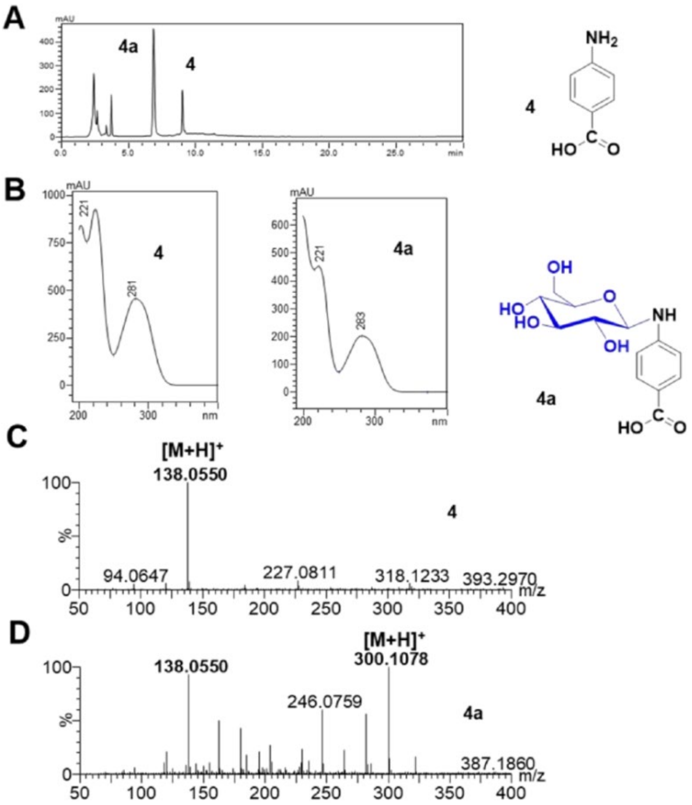

Figure S7. HPLC−PDA and HR−QTOF ESI/MS analysis conversion of 4-aminobenzoic acid (**4**) as an aglycon acceptor of YdhE in *C. glutamicum*. (**A**) HPLC chromatogram; (**B**) UV spectra of **4** and glucosylated product **4a**; (**C**) Selected ion chromatogram at m/z 138.0550 for substrate **4** $[M+H]^+$ and (**D**) Selected ion chromatogram at *m/z* 300.1078 for glucosylated product of **4** $[M+H]^+$.

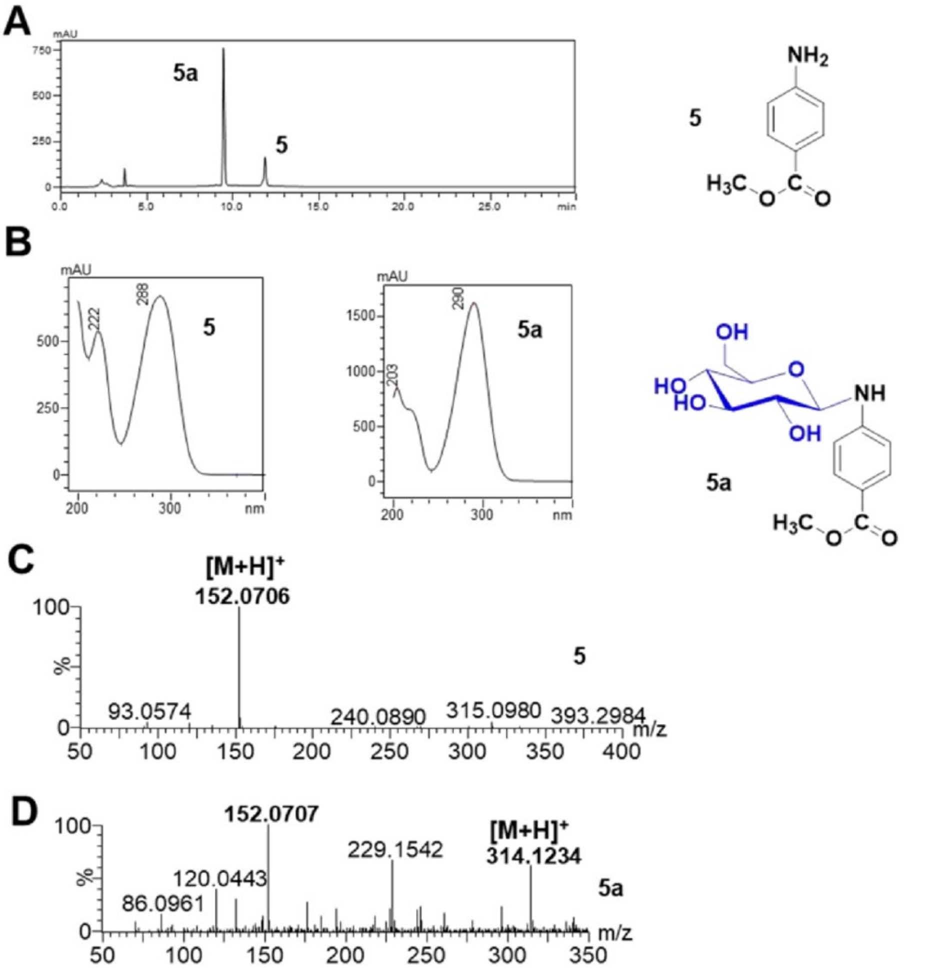

Figure S8. HPLC−PDA and HR−QTOF ESI/MS analysis conversion of methyl-4-aminobenzoate (**5**) as an aglycon acceptor of YdhE in *C. glutamicum*. (**A**) HPLC chromatogram; (**B**) UV spectra of **5** and glucosylated product **5a**; (**C**) Selected ion chromatogram at *m/z* 152.0706 for substrate **5** $[M+H]^+$ and (**D**) Selected ion chromatogram at *m/z* 314.1234 for glucosylated product of **5** $[M+H]^+$.

A

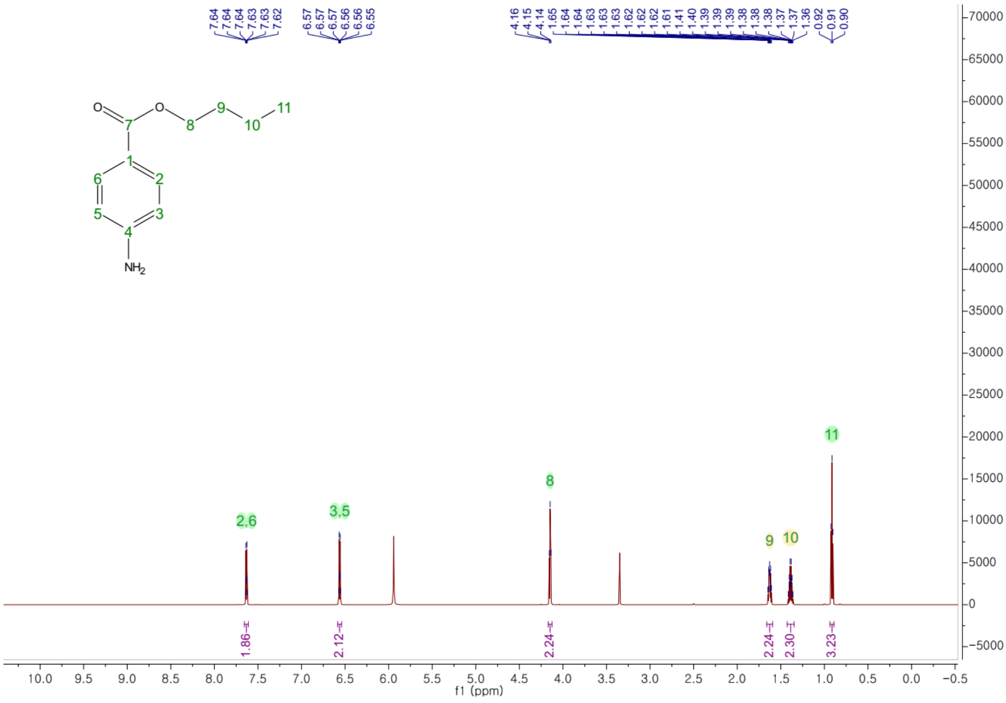

B

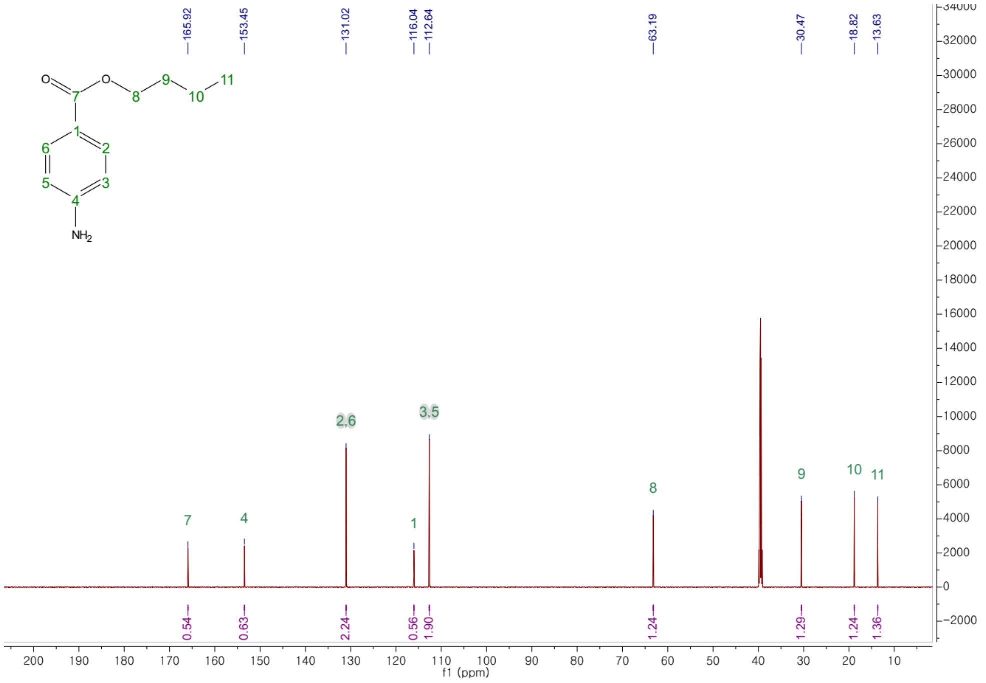

Figure S9. (**A**) ^{1}H NMR and (**B**) ^{13}C NMR of butyl-4-aminobenzoate standard.

A

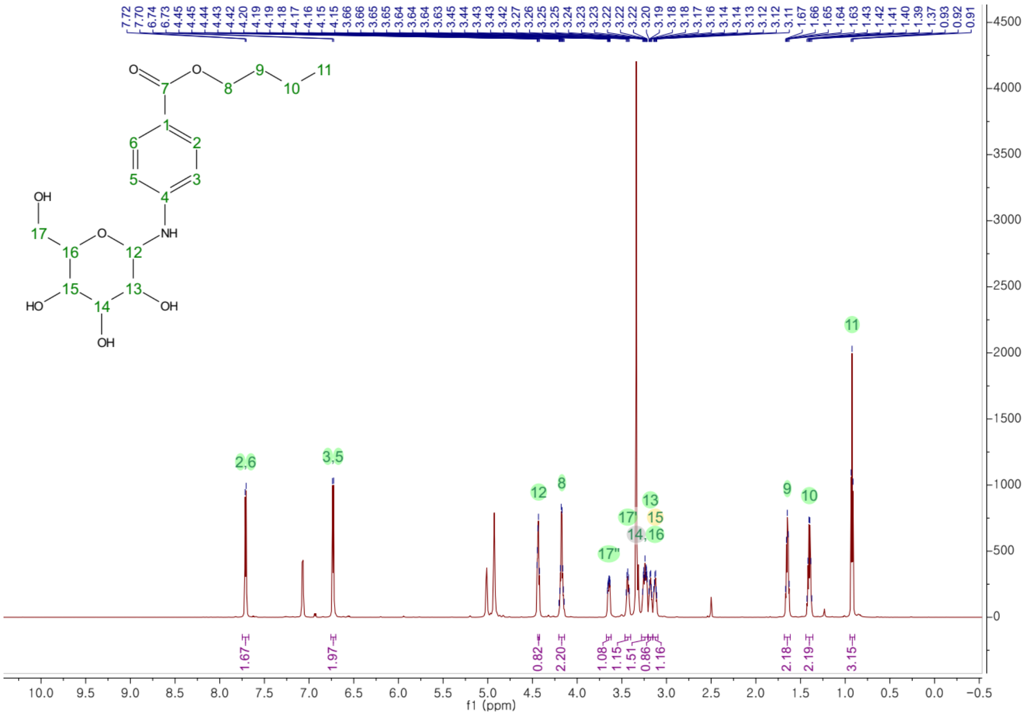

B

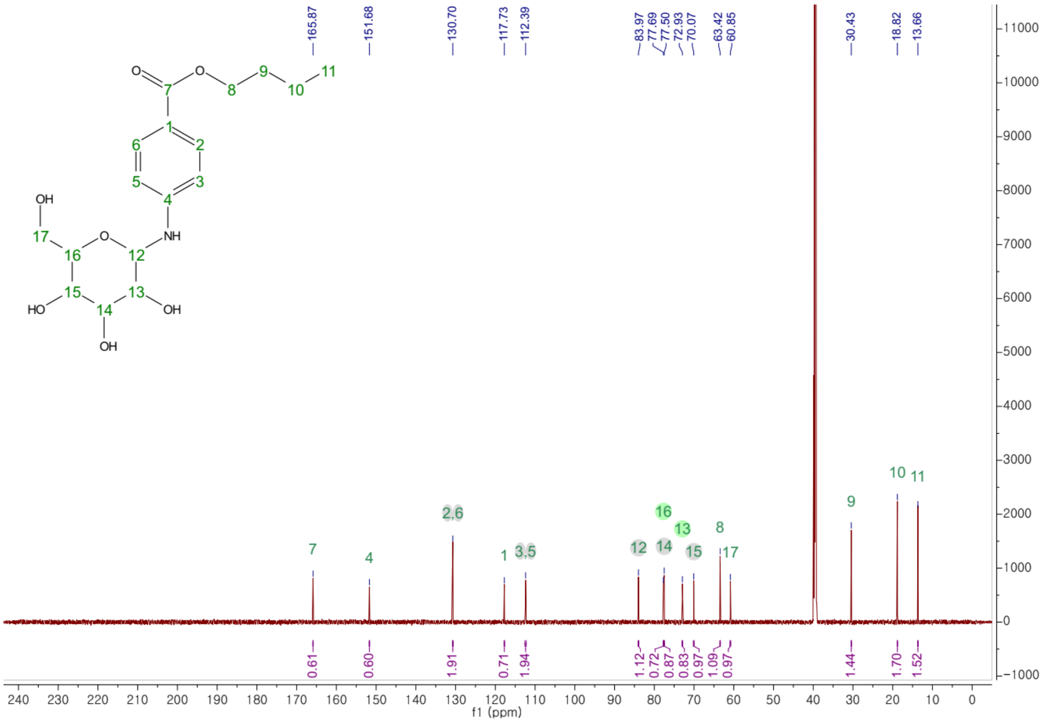

Figure S10. (**A**) ^{1}H NMR and (**B**) ^{13}C NMR of butyl-4-aminobenzoate-N-β-D-glucopyranoside.

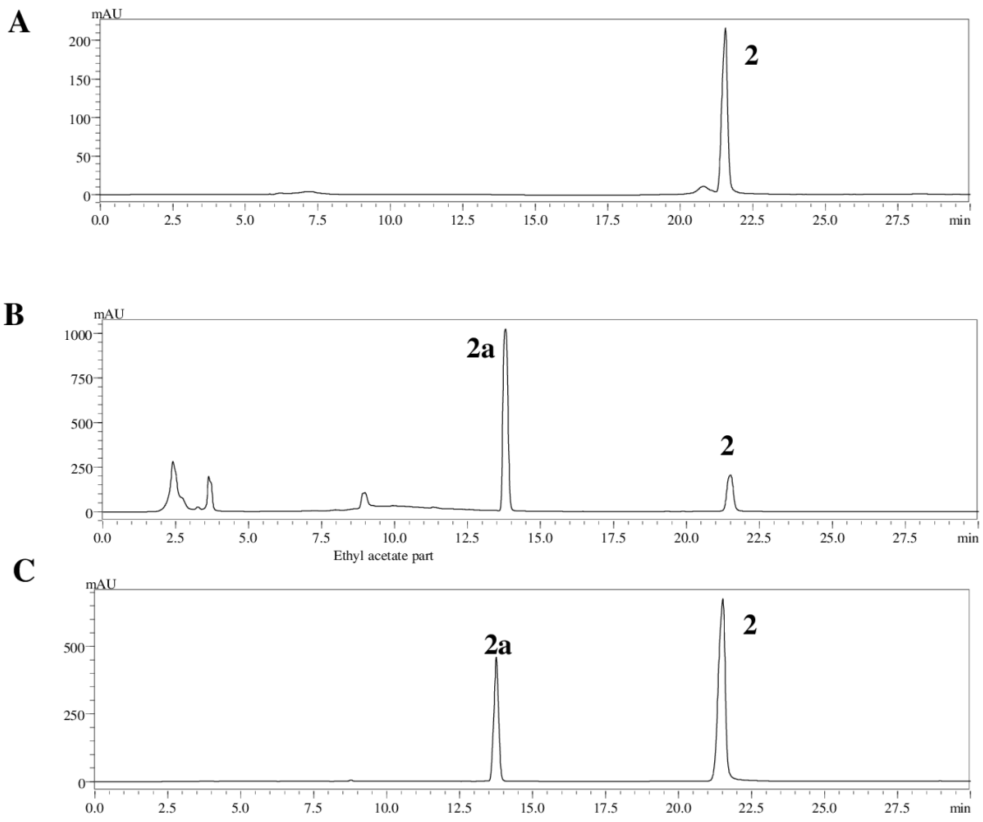

Figure S11. Water solubility test of butyl 4 aminobenzoate. (**A**) Standard butyl-4-aminobenzoate; (**B**) butyl-4-aminobenzoate (**2**) and its glucoside product (**2a**) in Aqueous (water) layer fraction and (**C**) butyl-4-aminobenzoate (**2**) and its glucoside product (**2a**) in ethyl acetate fraction

Table S1. List of sequences of *pTac* promoter and CO−YdhE gene using in *C. glutamicum* Note: Bold nucleotides signifiesrestriction enzymes recognition sites; italic sequences represents ribosome binding sites (RBS) and spacer.

NAME	SEQUENCE (5'-3')
*Spe*I−*pTac*−*RBS*−*Bam*HI	**ACTAGT**TTGACAATTAATCATCGGCTCGTATAATG<br>*AAGGAGATATACA***GGATCC**
CO-YdhE	ATGATCATGAAGAACATCCTGATCGTGAACTTCCCAGCCGAAGGCCACGTGAACCCAACC<br>CTGGGCATCACCAAGGCCTTCGCCGATCGCGGCGATAACGTGCACTACCTGTCCACCGAA<br>AAGTACAAGGATCGCCTGGAAGGCGTGGGCGCAACCGTGCACCTGTACAAGGACCTGGTG<br>CGCAACGCCCACATCGACCCAAACTCCCCATCCGGCCTGCTGGAATTCCTGAAGATCCAC<br>CTGAAGACCTCCCTGTACATCCTGGATATCGTGAAGGAACTGTCCAAGTCCATCTCCTTCG<br>ATGTGGTGTACTACGATACCTTCGGCGCCGGCGAACTGGTGCGCGATTACCTGAACATCCC<br>AGGCATCGCCTCCTCCGCATCCTTCCTGTTCGGCCAGGAACACAAGAAGATCCTGCCACTG<br>CACCCAGACTCCGGCGCCGAACTGCACCTGGACAAGGAATGCGAAGACCTGCTGGCCGAA<br>CTGAAGGAAAAGTACGGCGTGTCCCCACGCCACACCGGCCAGTTCATGTCCAACCAGGCC<br>GAACTGACCGTGGTGTACACCTCCCGCTACTTCCAGCCAGATTCCGGCCGCTTCGGCGACG<br>ACGTGCTGTTCATCGGCCCACGCTTCCCAAAGCGCCTGGATAAGACCGATTTCCCAGTGGA<br>ATCCCTGAAGAACGAAAAGGTCATCTATATCTCCATGGGCACCGTGCTGGGCAAGACCGC<br>CGATTTCTTCAACATGTGCATCGATGCCTTCCGCGATTTCGACGGCAAGGTGGTCATTGCA<br>GCCGGCGAAAAGTCCGATTACGCAGAAATCAAGGAAGTGCCAGAACACTTCATCATCGCC<br>CCATACGTGCCACAGCTGGAAGTGCTGAAGGAAGCAGACGTGTTCATCACCCACGGCGGC<br>ATGAACTCCGTGAACGAAGGCATCCACTACCGCGTGCCAATGGTGGTGCTGCCACACGAC<br>AAGGATCAGCCAATGATCGCCCAGCGCCTGAAGGAACTGAACGCCGGCTACCCACTGTTC<br>GCCGAAGAAGTGAACGCAGAACGCCTGCGCGACGCAGCCGAACAGGTGCTGACCGATGG<br>CAAGTACCAGGAAGGCATCCAGAAGATCGATGAATCCTTCTCCAACTGCATGGATATCAA<br>GGATGCCCTGGCACGCATCGATGAATACACCGCCCGCAAGAAGGTGGCAGCAGCCATCAC<br>CGAATCCCGCTACTAA

Table S2. Primers used in this study. Note: Bold nucleotides signifiesrestriction enzymes recognition sites.

NAME	SEQUENCE (5'-3')

F_*Nco*I_LacI	AG**<u>CCATGG</u>**ATGGCGGAGCTGAATTACATTC
R_*Nco*I_LacI	AA**<u>CCATGG</u>**TCAAGCCTTCGTCACTGGTCC
F_Ncgl2772	CATGCCTGACGACACTAAC
R_Ncgl2772	CGGCGGCGTCAGCGTCGATGC
F_CO-YdhE	CTGGAATTCCTGAAGATCC
R_CO-YdhE	CTGGCCGAACAGGAAGGATG

Table S3. List of substrates using in this study; (+): conversion and (-): no conversion.

	Substrate	Conversion
**1**	4-aminoazobenzene	+
**2**	butyl-4-aminobenzoate	+
**3**	4-aminosalycylic acid	+
**4**	4-aminobenzoic acid	+
**5**	methyl-4-aminobenzoate	+
**6**	3-(methylthio)aniline	-
**7**	3'-aminoacetophenone	-
**8**	3-amino-9-ethylcarbazole	-
**9**	2-aminobenzophenone	-
**10**	3-amino-N-phenylbenzamide	-
**11**	3-amino-4-ethoxy-benzaanilide	-
**12**	2-aminoazotoluene	-

Table S4. ^{1}H NMR and ^{13}C NMR data of butyl-4-aminobenzoate standard and butyl-4-aminobenzoate-N-*β*-D-glucopyranoside.

No	butyl-4-aminobenzoate (DMSO-d_6)		butyl-4-aminobenzoate-N-*β*-D-glucopyranoside (DMSO-d_6)		Intensities
	^{13}C	^{1}H	^{13}C	^{1}H	
1'			83.97	4.43 (d) *J* = 12.4 Hz	1H
2'			72.93	3.19 (dd) *J* = 8.5, 5.0 Hz	1H
3'			77.50	3.23 (m)	1H
4'			70.07	3.13 (dd) *J* = 9.2, 5.1 Hz	1H
5'			77.69	3.23 (m)	1H
6'			60.85	3.65 (ddd) *J* = 11.9, 5.5, 2.3 Hz (Sugar H-6′a)	1H
				3.43 (dt) *J* = 11.8, 5.9 Hz (Sugar H-6′b)	1H
1	116.04		117.73		
2	131.02	7.63 (m)	130.70	7.71 (d) *J* = 8.5 Hz	2H
3	112.64	6.56 (m)	112.39	6.73 (d) *J* = 8.4 Hz	2H
4	153.45		151.68		
5	112.64	6.56 (m)	112.39	6.73 (d) *J* = 8.4 Hz	
6	131.02	7.63 (m)	130.70	7.71 (d) *J* = 8.5 Hz	
7	165.92		165.87		
8	63.19	4.15 (t) *J* = 6.6 Hz	63.42	4.17 (m)	2H
9	30.47	1.63 (p) *J* = 6.6Hz	30.43	1.65 (p) *J* = 6.8 Hz)	2H
10	18.82	1.39 (m)	18.82	1.40 (h) *J* = 7.4 Hz	2H

11	13.63	0.91 (t) J = 7.4 Hz	13.66	0.92 (t) J = 7.4 Hz	3H
